# Mora - Automatic Generation of Moment-Based Invariants

**DOI:** 10.1007/978-3-030-45190-5_28

**Published:** 2020-03-13

**Authors:** Ezio Bartocci, Laura Kovács, Miroslav Stankovič

**Affiliations:** 8 Johannes Kepler University, Linz, Austria; 9 University of Birmingham, Birmingham, UK; 10 TU Wien, Vienna, Austria; 11 Chalmers, Gothenburg, Sweden

## Abstract

We introduce Mora, an automated tool for generating invariants of probabilistic programs. Inputs to Mora are so-called Prob-solvable loops, that is probabilistic programs with polynomial assignments over random variables and parametrized distributions. Combining methods from symbolic computation and statistics, Mora computes invariant properties over higher-order moments of loop variables, expressing, for example, statistical properties, such as expected values and variances, over the value distribution of loop variables.

## 1 Introduction

Probabilistic programs (PPs) are becoming more and more commonplace. Originally employed in randomized algorithms and cryptographic/privacy protocols, now gaining momentum due to the several emerging applications in the areas of machine learning and AI [5]. By introducing randomness into the program, program variables can no longer be treated as having single values; we must think about them as distributions. Dealing with distributions is much more challenging and some simplifications are required. Existing approaches, see e.g. [1,3,7,9,10], usually take into consideration only expected values or upper and lower bounds over program variables, or rely on user guidance for providing templates and hints.

One of the main challenges in analyzing PPs and computing their higher-order moments comes with the presence of loops and the burden of computing so-called *quantitative invariants* [7]. Quantitative invariants are properties that are true before and after each loop iteration and are crucial for analyzing the behavior of PP loops.

In this paper, we introduce the Mora tool for computing quantitative invariants of a class of PPs, called *Prob-solvable loops* [2], with random assignments, parametrized distributions, and polynomial probabilistic updates. Our implementation is available at:

https://github.com/miroslav21/mora, and successfully evaluated on a number of challenging examples. Unlike other existing approaches, e.g. [1,3,7,9], Mora computes non-linear invariants in a fully automatic way, without relying on user-provided templates/hints. The proposed automatic approach can handle an arbitrary number of loop iterations and also infinite loops. On the contrary, tools like PSI [4] support only the automatic analysis of probabilistic programs with a specified number of loop iterations.

This research was supported by the ERC Starting Grant 2014 SYMCAR 639270, the Wallenberg Academy Fellowship 2014 TheProSE, and the Austrian FWF project W1255-N23.

```
x=0
while true:
  u = RV(uniform, 0, b)
  g = RV(gauss, 0, 1)
  x = x - u @ 1/2; x + u @ 1/2
  y = y + x + g
```

Loop conditions are ignored, yielding non-deterministic PPs. The value of the random variable `u` is sampled by a uniform distribution with support in the real interval `[0, b]`, whereas the value of `g` is a random number from a normal distribution with mean (first moment) 0 and variance (second moment) 1. Updates to variable `x` are probabilistic: with probability `1/2`, the variable `x` is updated by `x-u`. Similarly, with probability `1/2`, `x` is updated by `x+u`. Further, updates to `u` and `g` do not depend on other variables; the update to `x` depends only on itself and `u`.

**Fig. 1.** An illustrative example of a Prob-solvable loop.

Moreover, the invariants inferred by MORA are not restricted to expected values but are quantitative invariants over the higher-order moments of program variables. We refer to such invariants as *moment-based invariants* [2]. To the best of our knowledge, no other approach can so far automatically compute higher-order moments of PPs, not even for the restricted yet expressive enough class of Prob-solvable loop.

The purpose of this paper is to describe what MORA can do and how it can be used. The paper is intended as a tool demonstration and guide for potential users of MORA. We focus on the usage and implementation aspects of MORA. For details on theoretical foundations and algorithmic aspects of MORA for computing moment-based invariants, we refer to [2]. We note however that, when compared to the experimental setup of [2], MORA comes with a completely new design, fully implemented in `python` and supporting an easy installation and use by even non-experts in PPs.

## 2 MORA– Programming Model

Input programs to MORA are PP loops that are Prob-solvable [2]. In Figure 1, we give an example of a Prob-solvable loop and use this example as a running example to guide the potential users of MORA in the rest of this paper.

In a nutshell, the probabilistic assignments of Prob-solvable loops involve (i) variable values drawn from random distributions, such as uniform or normal distributions, and (ii) random variable updates. In the sequel, we write `RV` to refer to a random variable. Input programs to MORA thus satisfy the following two properties:

(1) Input programs to MORA are PPs generated from the grammar in Figure 2.

(2) In addition to the grammar of Figure 2, MORA requires its PP input to be Prob-solvable, imposing further restrictions as follows:

- PP loop variables are different from each other and from parameters;
- probabilities used within a variable update sum up to 1;
- updated variables depend on themselves linearly and may depend polynomially only on other variables that have been previously updated.

Note that Figure 1 satisfies all constraints above, and thus is Prob-solvable.

Grammar defining PP inputs to MORA

```
PROGRAM → INIT_ASSIGNS "while true:" RV_ASSIGNS UPD_ASSIGNS

INIT_ASSIGNS → INIT_ASSIGN | INIT_ASSIGN INIT_ASSIGNS
RV_ASSIGNS → RV_ASSIGN | RV_ASSIGN RV_ASSIGNS
UPD_ASSIGNS → UPD_ASSIGN | UPD_ASSIGN UPD_ASSIGNS

INIT_ASSIGN → VAR " = " INIT_EXPR
RV_ASSIGN → VAR " = " RV_EXPR
UPD_ASSIGN → VAR " = " UPD_BRANCHES

UPD_BRANCHES → UPD_BRANCH | UPD_BRANCH UPD_BRANCHES
UPD_BRANCH → UPD_EXPR "@" UPD_PROB
UPD_PROB → SIMP_EXPR

INIT_EXPR → RV_EXPR | SIMP_EXPR
RV_EXPR → "RV(uniform, " SIMP_EXPR ", " SIMP_EXPR ")"
            | "RV(gauss, " SIMP_EXPR ", " SIMP_EXPR ")"
UPD_EXPR → UPD_EXPR OP UPD_EXPR | VAR | ATOM
SIMP_EXPR → SIMP_EXPR OP SIMP_EXPR | ATOM

ATOM → NUM | PARAMETER
OP → [*+-]
VAR → [a-zA-Z][a-zA-Z0-9]*
PARAMETER → [a-zA-Z][a-zA-Z0-9]*
NUM → [-]?[0-9]+[.]?[0-9]*([\/][1-9][0-9]*)?
```

**Fig. 2.**

## 3 MORA– Usage

We describe the easiest way MORA can be used to generate moment-based invariants:

- Save a Prob-solvable loop to a file, for example save Figure 1 in the file `running`
- In the main MORA folder invoke `python` with `python3.7` and execute:

```
from mora.mora import mora
```

- Run MORA using the command:

```
mora("running", goal=GOAL),
```

where `GOAL` can be (i) a specific natural number $k \geq 1$, in which case MORA computes the $k$th moments of all variables from `running`; (ii) a specific moment of one loop variable of `running` (e.g. `"x^2"` specifying the second moment of a variable `x` of Figure 1); or (iii) a list containing the goals as just specified. One can specify finitely many goals as inputs to MORA; yet, at least one goal is required. For example, by running `mora("running", [1, "x^2", "x^3"])`, MORA computes the expected values (first moments, i.e. 1) of all variables from Figure 1, as well as the second and third moments of variable `x` of Figure 1 (specified by `x^2` and `x^3`, respectively).

MORA is completely automatic. That is, once an execution of MORA is started on a given Prob-solvable loop and input goals, MORA outputs the higher-order moments, and thus moment-based invariants, of its loop w.r.t. the specified input goals. To this end, MORA computes the expected values of all monomials over loop variables, on which one of the goals from `Goal` depends. In general, computing the `k`th moment requires computing the expected values of all monomial expressions over loop variables, such that the total degree of the monomials is less or equal than $k$ – see [2] for more details.

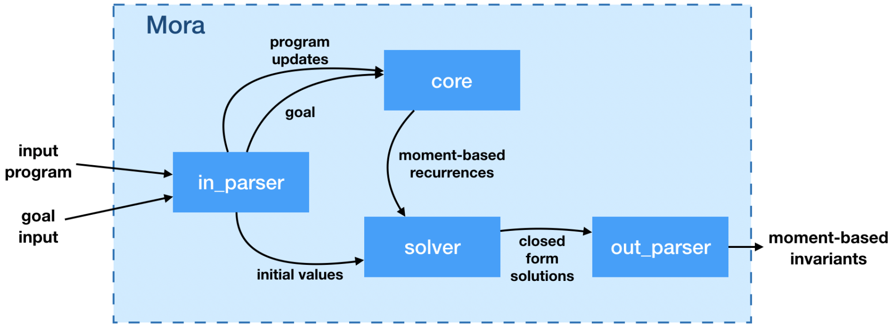

**Fig. 3.** MORA workflow diagram.

In the rest of the paper, we will illustrate the main steps of MORA, by considering Figure 1 as its input loop and `[1, 2]` as its list of input goals. With such an input goal, MORA is set to compute the first and second moments of each variable of Figure 1. Note, that even if `1` was omitted from the aforementioned input goal, MORA would still need to compute some of the first moments of the variables, as they are required for computing the second-order moments. In the sequel, we show-case the MORA behaviour for:

$$\texttt{mora("running", [1, 2]).} \tag{1}$$

## 4 MORA– Tool Overview

We first give details on our implementation. We then present the overall workflow of MORA in Figure 3, based on which we overview the main components of our tool.

***Overall Implementation.*** MORA is implemented in `python3`, requiring `python` version of at least 3.7. MORA relies on the `diofant` and `scipy` libraries: (i) the `python` library `diofant` is used in MORA for symbolic mathematical computations and recurrence solving; (ii) the `scipy` library, and in particular its statistics module `scipy.stats`, is used in MORA to handle probability distributions and statistical functions, as well as to simplify and compute expressions involving probability distributions and initial values of variables. Altogether, our implementation comprises of around 350 lines of code.

**MORA –** ***Parser.*** MORA first checks whether a given input program is Prob-solvable, by checking the requirements of Section 2. If the input program is not Prob-solvable, an error is reported, and the execution of MORA stops. Otherwise, within its parser module, MORA extracts initial values from its input loop, rewrites loop updates into equations over expected values of monomial expressions over loop variables, and processes the list of its input goals to identify which higher-order moments need to be computed.

For our demo execution (1), MORA extracts the initial value `x(0)=0`, where `x(0)` denotes the initial value of `x` before the loop. Using the input goals specified in (1), MORA is set to compute the expected values of {`u, g, x, y, u^2, g^2, x^2,`

y^2 characterizing the first and second moments of all loop variables of Figure 1. Further, the loop updates of Figure 1 are rewritten by MORA into equations over expected values, as follows:

$$\begin{cases} E[x^k(n+1)] = E[1/2 \cdot (x(n) - u(n+1))^k + 1/2 \cdot (x(n) + u(n+1))^k] \\ E[y^k(n+1)] = E[(y(n) + x(n+1) + g(n+1))^k] \end{cases}, \quad (2)$$

where $n \geq 0$ is the loop counter of Figure 1, $x(n)$ denotes the value of x at the $n$th loop iteration, and $E[expr]$ is the expected value of an expression $expr$.

**MORA – *Core*.** After rewriting probabilistic loop updates into equations over expected values, MORA rewrites these equations into non-probabilistic recurrences over so-called E-variables, with the loop counter $n$ being the recurrence index. E-variables are simply variables created from monomials over original variables. Thanks to the restrictions defining PPs to be Prob-solvable, the resulting recurrences are linear recurrences with constant coefficients, that is C-finite recurrences, whose closed forms can always be computed [8]. MORA solves these recurrences by calling its *Solver* module.

Using the equations (2) over expected values, the non-probabilistic recurrences of Figure 1 generated by MORA are as follows, using the MORA synthax:

```
y = x + y
g ∗ ∗2 = 1
x = x
u = b/2
x ∗ ∗2 = b ∗ ∗2/3 + x ∗ ∗2                      (3)
u ∗ ∗2 = b ∗ ∗2/3
y ∗ ∗2 = b ∗ ∗2/3 + x ∗ ∗2 + 2 ∗ x ∗ y + y ∗ ∗2 + 1
g = 0
x ∗ y = b ∗ ∗2/3 + x ∗ ∗2 + x ∗ y
```

The left-hand sides of these equations represent values of E-variables at iteration $n+1$, while monomials over original variables on the right-hand side represent E-variables at iteration $n$. For example, the first equation of (3) stands for $E[y(n+1)] = E[x(n)] + E[y(n)]$. On the other hand, the fourth equation of (3) represents $E[x(n+1)^2] = \frac{b^2}{3} + E[x(n)^2]$, as $b$ is a constant parameter and x**k in python denotes the $k$th power of $x$.

**Solver.** In this module, MORA extracts and solves recurrences from the non-probabilistic equations over E-variables computed by its *Core* module. By exploiting the structure of Prob-solvable programs, MORA also optimizes the order in which recurrences are solved, e.g. independent recurrences are solved first. Partial solutions can be used to reduce the complexity of the latter recurrences. MORA then uses the diofant library to handle and solve single recurrences.

For Figure 1, using the E-variable equations of (3), the following closed form solutions are computed by MORA:

$$\begin{aligned}
&E[u^2] = \frac{b^2}{3}\\
&E[x^1] = 0\\
&E[y^1] = y(0)\\
&E[x^2] = \frac{b^2 n}{3}\\
&E[u^1] = \frac{b}{2}\\
&E[y^1 x^1] = \frac{b^2 n}{6}(n+1)\\
&E[y^2] = \frac{n}{18}\left(2b^2n^2 + 3b^2n + b^2 + 18\right) + y(0)^2\\
&E[g^1] = 0\\
&E[g^2] = 1
\end{aligned} \tag{4}$$

with $y(0)$ standing for the initial value of $y$ (treated as a parameter, since not specified).

**MORA – *Out_Parser.*** MORA's output consists of basic information about the program and the goal, moment-based invariants computed, and computation time. By default, the MORA output is shown only on the screen. However, an optional argument can specify if an output file should be created. Two possible values for `output_format` are (i) `"txt"`, producing a simple human-readable file, and (ii) `"tex"`, producing a file with invariants in LaTeX format (as given in (4) above).

## 5 Evaluation

A proof-of-concept implementation, together with initial experiments, were already given in our work on generating moment-based invariants [2]. MORA comes however with a new design and re-implementation of [2], significantly improving the experimental setting and evaluations of [2]. Table 1 compares MORA against the experiments of [2], on a subset of Prob-solvable loops from [2], evidencing that MORA is faster than our initial proof-of-concept implementation. This is due to the following reasons:

- MORA now optimizes the order in which recurrences are sent to the `diofant` recurrence solver. This reduces the amount of necessary symbolic computation and speeds up the process.
- While MORA is implemented entirely in `python`, with limited usage of external libraries, the previous implementation was done in `Julia` and relied on calls to the `sympy` library of `python`.
- MORA does not rely on `Aligator` [6] for handling systems of recurrences, allowing us to eliminate some intermediate and redundant steps.

| Program | Moment | Runtime PoC ($s$) | Runtime MORA ($s$) |
|---|---|---|---|
| SUM_RND_SERIES | 1 | 0.31 | 0.22 |
| | 2 | 2.89 | 0.93 |
| | 3 | 17.7 | 2.47 |
| STUTTERINGA | 1 | 0.44 | 0.25 |
| | 2 | 2.20 | 1.07 |
| | 3 | 8.48 | 3.35 |
| STUTTERINGC | 1 | 1.80 | 0.66 |
| | 2 | 72.5 | 12.2 |
| | 3 | 2144 | 73.9 |
| SQUARE | 1 | 0.38 | 0.22 |
| | 2 | 2.46 | 0.73 |
| | 3 | 8.70 | 1.67 |

**Table 1.** Comparison of MORA vs. proof-of-concept (PoC) implementation of [2].

## 6 Conclusion

We described MORA, a fully automated tool for generating invariants of probabilistic programs. MORA combines recurrence solving, symbolic summation and statistical reasoning, and derives higher-order moments of loop variables in probabilistic programs.

## References

[1] Barthe, G., Espitau, T., Fioriti, L.M.F., Hsu, J.: Synthesizing Probabilistic Invariants via Doob’s Decomposition. In: CAV. LNCS, vol. 9779, pp. 43–61. Springer (2016)

[2] Bartocci, E., Kovács, L., Stankovic, M.: Automatic generation of moment-based invariants for prob-solvable loops. In: Proc. of ATVA 2019: the 17th International Symposium on Automated Technology for Verification and Analysis. LNCS, vol. 11781, pp. 255–276 (2019)

[3] Chakarov, A., Sankaranarayanan, S.: Expectation Invariants for Probabilistic Program Loops as Fixed Points. In: SAS. LNCS, vol. 8723, pp. 85–100 (2014)

[4] Gehr, T., Misailovic, S., Vechev, M.T.: PSI: Exact Symbolic Inference for Probabilistic Programs. In: CAV. LNCS, vol. 9779, pp. 62–83 (2016)

[5] Ghahramani, Z.: Probabilistic Machine Learning and Artificial Intelligence. Nature **521**(7553), 452–459 (2015) 10.1038/nature14541 26017444

[6] Humenberger, A., Jaroschek, M., Kovács, L.: Aligator.jl - A Julia Package for Loop Invariant Generation. In: CICM. LNCS, vol. 11006, pp. 111–117 (2018)

[7] Katoen, J.P., McIver, A.K., Meinicke, L.A., Morgan, C.C.: Linear-Invariant Generation for Probabilistic Programs: Automated Support for Proof-Based Methods. In: SAS. LNCS, vol. 6337, pp. 390–406 (2010)

[8] Kauers, M., Paule, P.: The Concrete Tetrahedron - Symbolic Sums, Recurrence Equations, Generating Functions, Asymptotic Estimates. Texts & Monographs in Symbolic Computation, Springer (2011)

[9] Kura, S., Urabe, N., Hasuo, I.: Tail Probabilities for Randomized Program Runtimes via Martingales for Higher Moments. In: TACAS. LNCS, vol. 11428, pp. 135–153 (2019)

[10] McIver, A., Morgan, C.: Abstraction, Refinement and Proof for Probabilistic Systems. Monographs in Computer Science, Springer (2005)

